# Supplementary material for: Lactobacillus supports Clostridiales to restrict gut colonization by multidrug-resistant Enterobacteriaceae
Source: Nat Commun. 2022 Sep 24;13:5617. doi: 10.1038/s41467-022-33313-w (PMC9509339; doi:10.1038/s41467-022-33313-w)
Supplement: Supplementary file 1 — Supplementary Information [file 41467_2022_33313_MOESM1_ESM.pdf]

SUPPLEMENTARY FIGURES

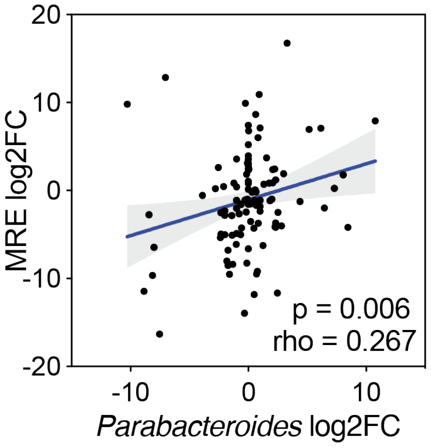

Supplementary Figure 1. Changes in *Parabacteroides* are positively associated with changes in MRE levels in acute leukemia patients (ALP). Log2 fold change (log2FC) in the levels of *Parabacteroides* as compared to the log2FC in the levels of MRE in pairs of consecutive samples collected from ALP. Two-sided Spearman correlation test, p=0.006, N=106 pairs of samples. The line represents the linear regression mean and the grey shadow is the 95% CI. Source data are provided as a Source Data file.

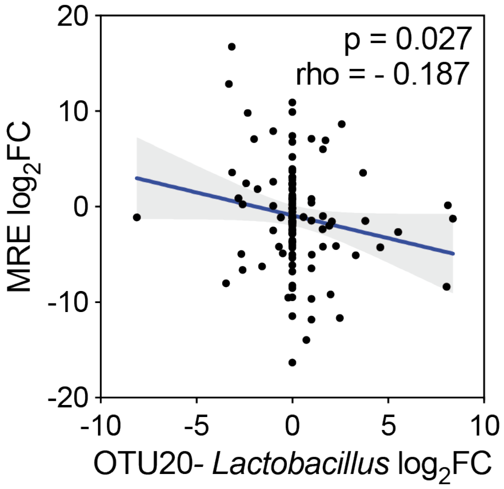

Supplementary Figure 2. Changes in the most abundant *Lactobacillus* OTU (OTU20) are negatively associated with changes in multidrug-resistant *Enterobacteriaceae* (MRE) levels in hospitalized patients. Changes in abundance of OTU20 in our patients cohort are negatively associated with changes in MRE levels. One-sided Spearman correlation test, p=0.027, rho=-0.187, N=106 pairs of samples. The line represents the linear regression mean and the grey shadow is the 95% CI. FC: fold change. Source data are provided as a Source Data file.

Study	Cohort	Country	N
This study	NA	Spain	47
Valles-colomer et al.	FGFP	Belgium	1054
Park et al.	MORINAGA	Japan	642
Latorre-Perez et al.	NA	Spain	530
Park et al.	NIBIOHN	Japan	954
Scepanovic et al.	MILIEU	France	1311

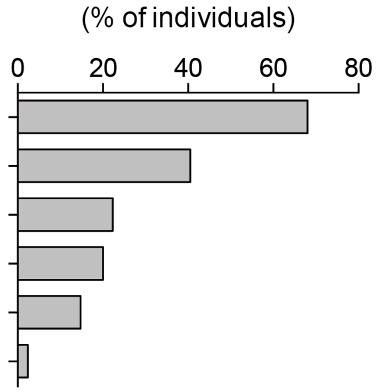

Supplementary Figure 3. Prevalence of the *Lactobacillus* OTU20 in different human cohorts. A representative sequence of the *Lactobacillus* OTU20 was aligned against 16S rRNA sequences from different study cohorts. The % of individuals containing sequences that could be assigned to the *Lactobacillus* OTU20 from our cohort (see methods) is represented with bars. N= Number of individuals included in the cohort. NA: only one cohort was included in that study and no specific name was given to that cohort. Source data are provided as a Source Data file.

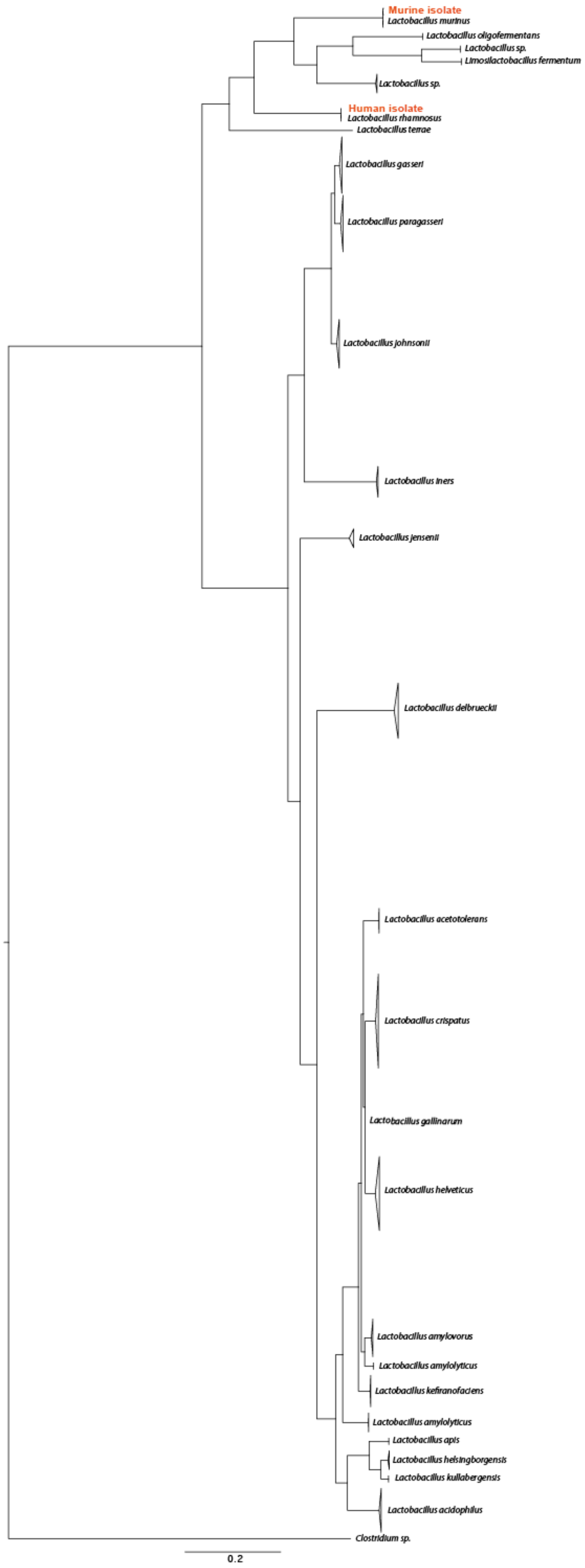

Supplementary Figure 4. Phylogenetic characterization of the *Lactobacillus* phylogeny and identification of the phylogenetic relationships of the two *Lactobacillus* isolates from this study. Phylogenetic tree was constructed with the core-genome of 115 *Lactobacillus* reference genomes plus the two isolates. To facilitate visualization, genomes belonging to the same species were grouped together and only the species name is shown. Red text indicates the two

Lactobacillus strains isolated in this study. The root of the tree was inferred using a phylogenetically distant bacterial species (*Clostridium cuniculi*). Source data are provided as a Source Data file.

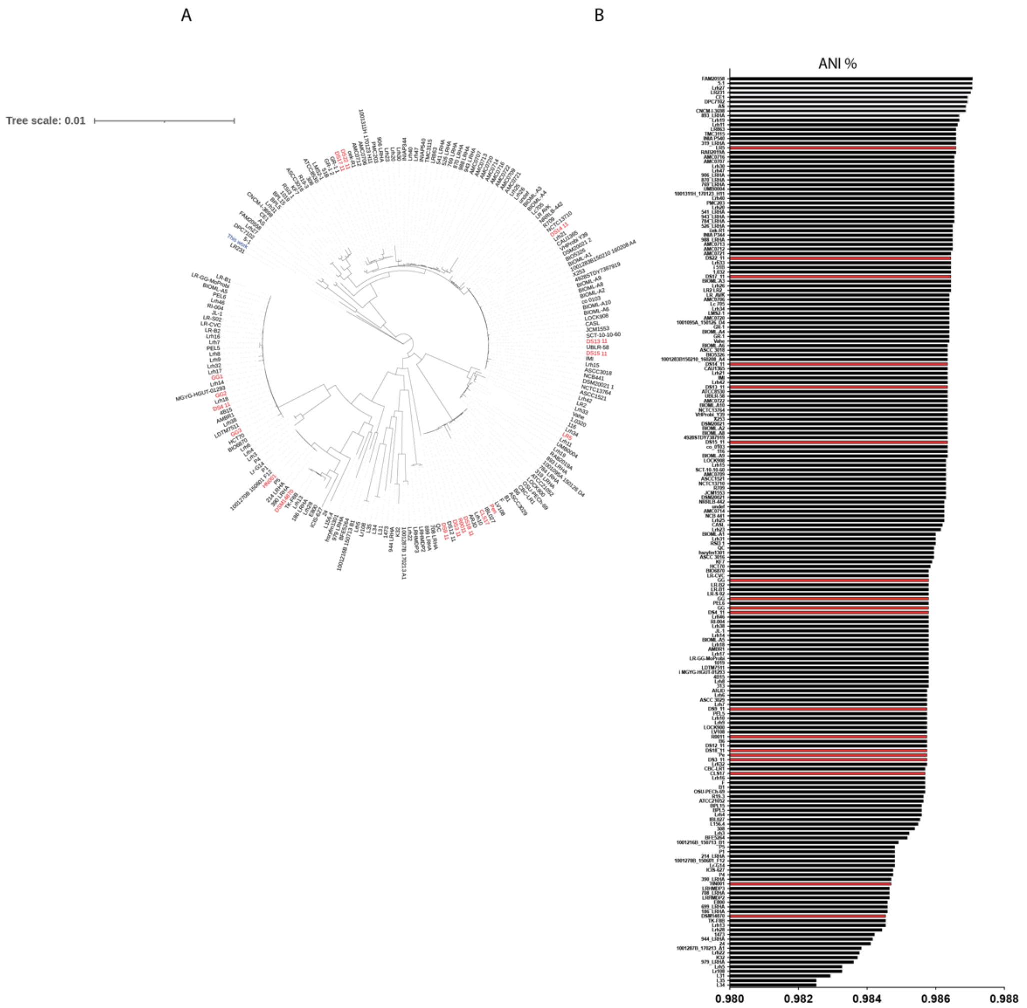

Supplementary Figure 5. Genome similarity of the *L. rhamnosus* isolate obtained in this study compared to other *L. rhamnosus* isolates. (A) Phylogenetic tree was constructed with the core-genome of 198 *L. rhamnosus* genomes deposited in NCBI plus the *L. rhamnosus* isolate from this study (blue). **(B)** Average nucleotide identity between the *L. rhamnosus* isolated in this study and *L. rhamnosus* genomes. Red bars and fonts indicate *L. rhamnosus* strains that have been tested as probiotics in humans (see Suppl. Data File 4). Source data are provided as a Source Data file.

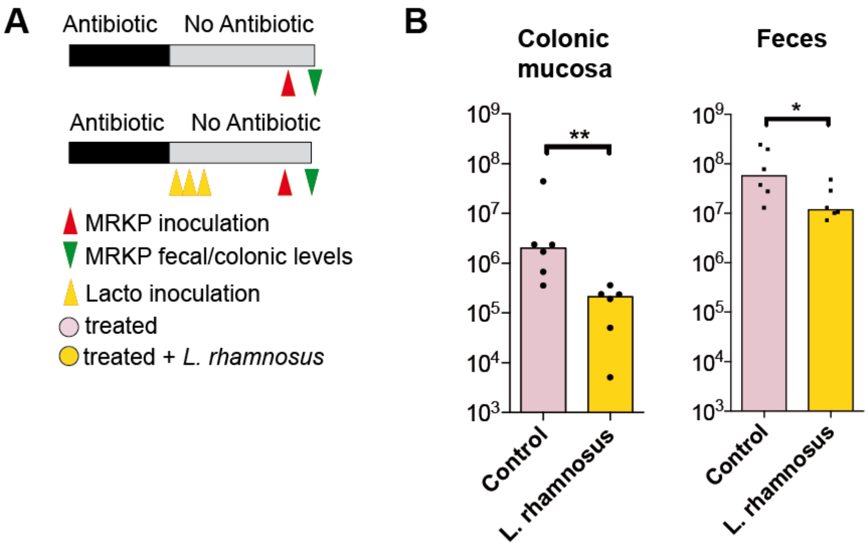

Supplementary Figure 6. *L. rhamnosus* restricts multidrug-resistant *K. pneumoniae* (MRKP) colonization of the colonic mucosa. (A) Schematic representation of the experimental model. Mice were treated with antibiotics (AVN) for one week in the drinking water. A group of mice received *L. rhamnosus* during 3 consecutive days by oral gavage, starting one day after stopping antibiotic treatment. A control group of mice received the bacterial vehicle instead (PBS-GC). Two weeks post antibiotic cessation, mice were orally gavaged with MRKP. **(B)** Two days after MRKP inoculation, mice were euthanized and the levels of MRKP were quantified in the colonic mucosa or in fecal samples. Two-sided Mann-Whitney test, *p=0.045, **p=0.004, N=6 mice per group. Bars represent the median. Source data are provided as a Source Data file.

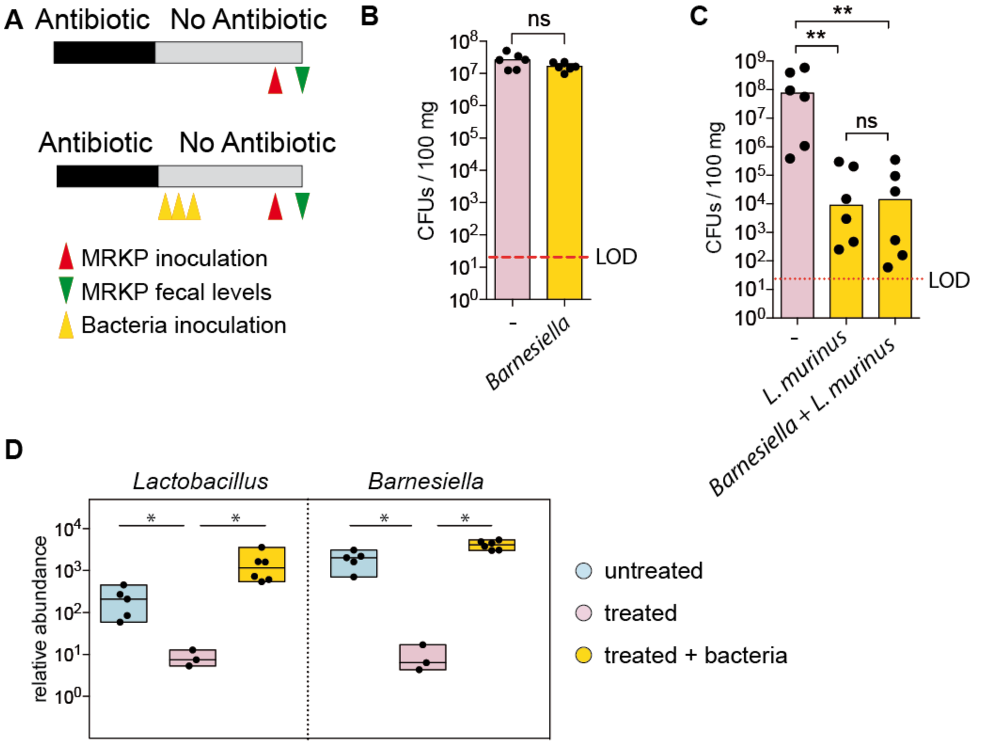

Supplementary Figure 7. Administration of *Barnesiella* does not restrict multidrug-resistant *K. pneumoniae* (MRKP) intestinal colonization. (A) Schematic representation of the experiment in which we tested the role of *Barnesiella* in restricting MRE intestinal colonization. Mice were treated with antibiotics (AVN) for a week in drinking water. After cessation of the treatment, one group was orally gavaged with specific commensal bacteria as depicted for three consecutive days, while another group received the bacterial vehicle (PBS-GC). Two weeks after the end of the antibiotic treatment, and 11 days after the last oral gavage of the bacteria, mice were inoculated with MRKP. **(B)** Administration of *Barnesiella* alone does not restrict MRKP colonization. Two-sided Mann-Whitney test, ns- not significant: p=0.24, N=6 mice per group. **(C)** Administration of *Lactobacillus* restricts MRKP intestinal colonization to the same extent as administration of *Barnesiella+Lactobacillus.* Two-sided Mann-Whitney test, **p<0.001, ns- not significant: p=0.818, N=6 mice per group. CFUs: colony forming units. **(D)** *Lactobacillus* and *Barnesiella* levels in untreated mice, mice treated with AVN (2 weeks after antibiotic cessation) or mice treated with AVN that received *Lactobacillus* and *Barnesiella.* Two-sided Mann-Whitney test, *p<0.05, N=5, 3 and 6 mice per group. Boxes extend from minimum to maximum. The line within the boxes represents the median. Bars represent the median. No adjustment for multiple comparisons was applied in the statistical analysis. Source data are provided as a Source Data file.

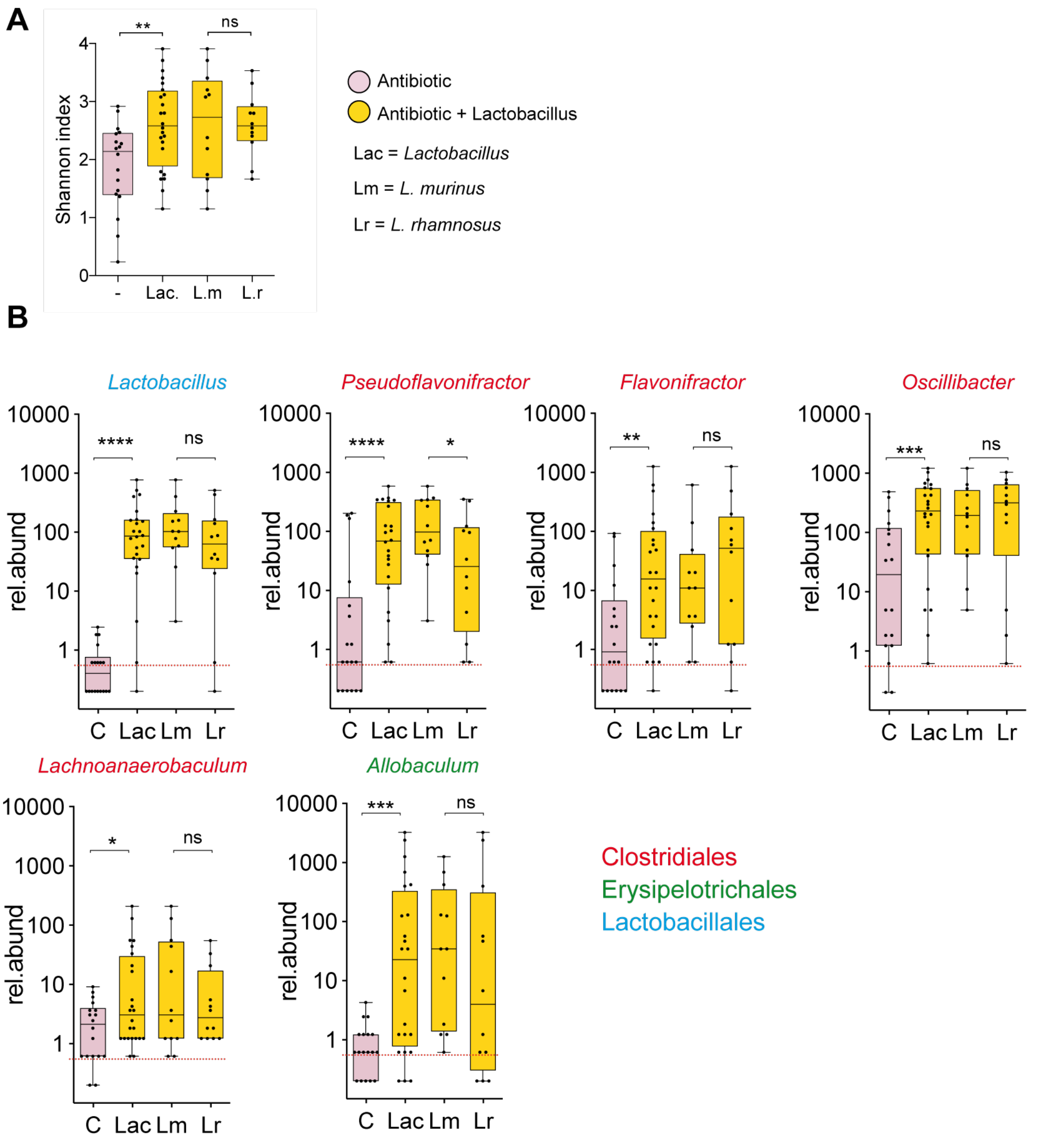

Supplementary Figure 8. Both *L. murinus* and *L. rhamnosus* promote the recovery of Clostridiales bacteria in antibiotic-treated mice. (A) Bacterial diversity increases after administration of *Lactobacillus* strains (*L. murinus* or *L. rhamnosus*) to AVN-treated mice and no differences are detected between *Lactobacillus* strains. Two-sided T-test, **p=0.004, ns- not significant: p=0.965, N=18, 24, 12 and 12 mice per group. **(B)** Statistically significant differences detected upon *Lactobacillus* administration among the most prevalent genera (median>0.01%). Comparison of the microbiota composition between mice that received *Lactobacillus* after AVN cessation and those that did not receive any bacterial strain revealed the recovery of the Clostridiales genera in mice gavaged with *Lactobacillus*. Minimal differences are detected between *Lactobacillus* strains. Only genera significantly different (p<0.05, q<0.1) between the control group and the group receiving *Lactobacillus* were analyzed. Two-sided Ancom2 test, ****p<0.0001,***p<0.001,**p=0.002,*p<0.05, ns- not significant: p>0.05, N=18, 24, 12 and 12 mice per group. Rel.abund: relative abundance: counts per 10000 sequences. Boxes extend from

the 25th to 75th percentiles. The line within the boxes represents the median. Whiskers indicate the maximum and minimum values. Source data are provided as a Source Data file.

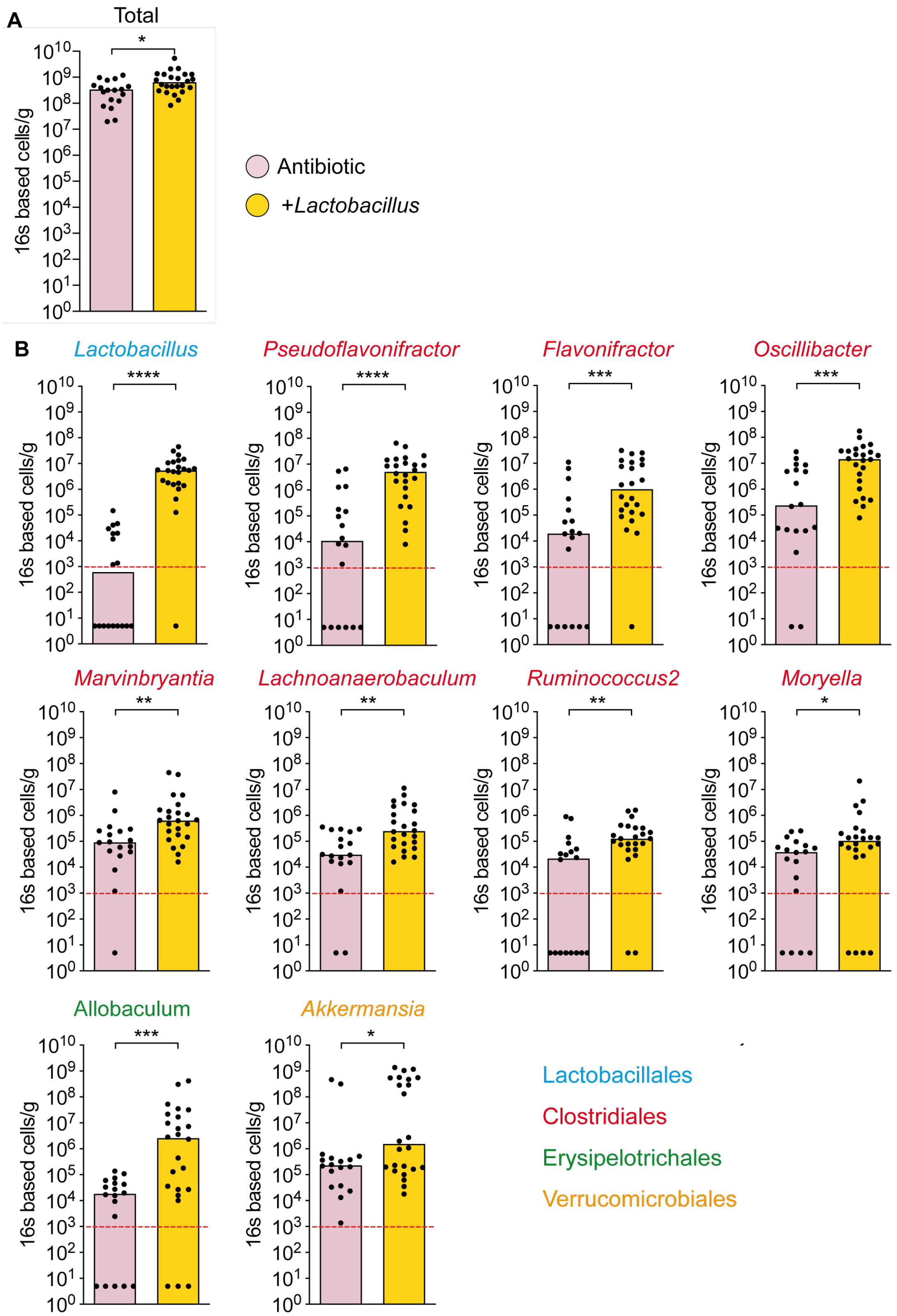

Supplementary Figure 9. ***Lactobacillus*** **increases the absolute abundances of specific genera, mainly from the order Clostridiales. (A)** Total numbers of fecal bacteria inferred from 16S rRNA qPCR data (see methods). Two-sided Mann-Whitney test, *p=0.012, N=18 and 24 mice per group. **(B)** Genera whose abundances significantly differ between the group of mice that received *Lactobacillus* and the group of mice that received the bacterial vehicle (PBS-GC). The abundances were calculated by normalizing the relative abundances with the total bacterial fecal numbers inferred from 16S rRNA qPCR data. Two-sided Mann-Whitney test, ****p<0.0001; ***p<0.001; **p<0.01, *p<0.05 and Benjamini and Hockberg correction (q<0.1), N=18 and 24 mice per group. p and q values for all analyzed genera and other bacteria that could not be classified to the genus level are shown in Suppl. Data File 7. Bars represent the median. Points below the red line represent samples in which a particular bacteria was not detected. Source data are provided as a Source Data file.

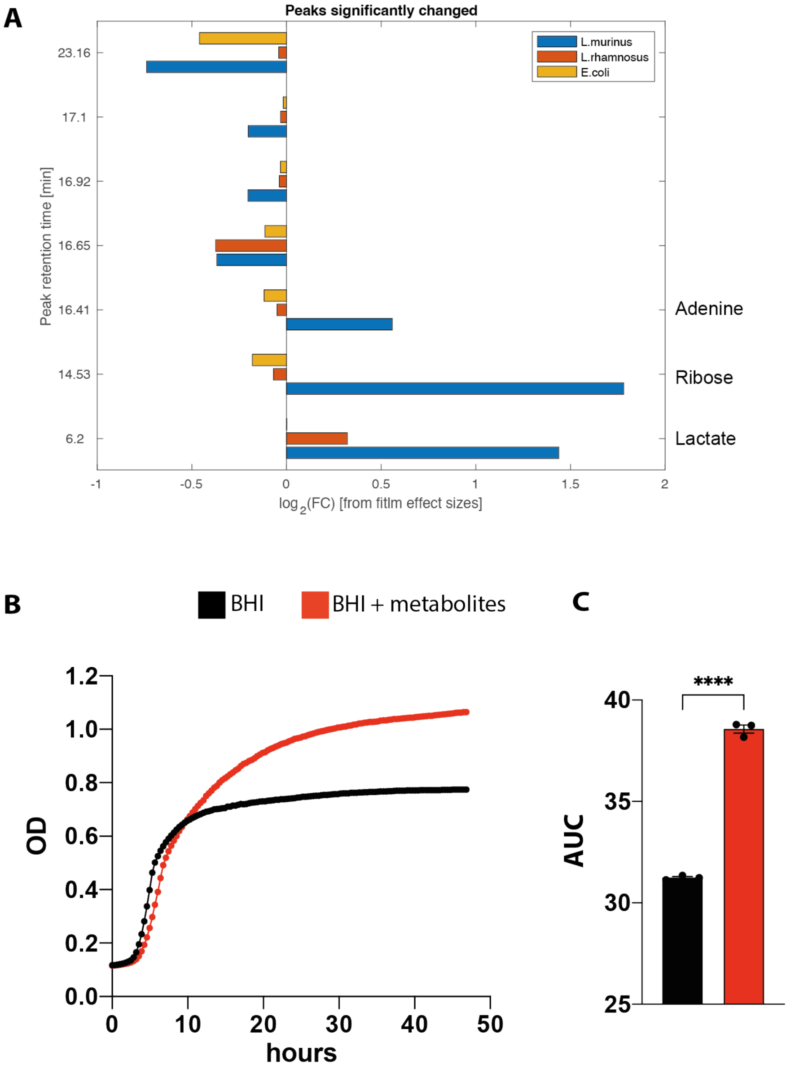

Supplementary Figure 10. *Lactobacillus* derived metabolites promote the growth of a Clostridiales strain *in vitro*. *L. murinus, L. rhamnosus* or *E. coli* were grown in BHI and metabolomics profile of their supernatants was analyzed. Linear mixed-effects model implemented in Matlab R2021b was used to calculate average log2 fold change (log2FC) between different species supernatants and BHI. **(A)** GC-MS identified peaks whose abundance changes (log2FC) in *Lactobacillus* conditioned media. Linear mixed-effects model comparing the abundance of a peak in a BHI culture with the abundance of that peak in un-inoculated BHI media, p<0.05, N=6 metabolomic analysed aliquots from each bacterial culture, exact p values can be found in Source Data file. No adjustment for multiple comparisons was applied in the statistical analysis. Bars represent the average log2FC in the abundance of significant peaks calculated with the linear mixed-effects model. Metabolites of interest (of those peaks that increased upon *Lactobacillus* growth) are shown. **(B)** *Flavonifractor plautii* was grown in BHI or BHI supplemented with metabolites identified in (A): adenine, ribose, lactate. OD: optical density. **(C)** Area under the growth curve (AUC) shown in (B). The growth of *F. plautii* was significantly increased by the administration of metabolites derived from *Lactobacillus* growth. Two-sided T-Test, ****p=2.4e-5, N=3 bacterial cultures for each group. Bars represent the mean, whiskers represent the SEM. Source data are provided as a Source Data file.

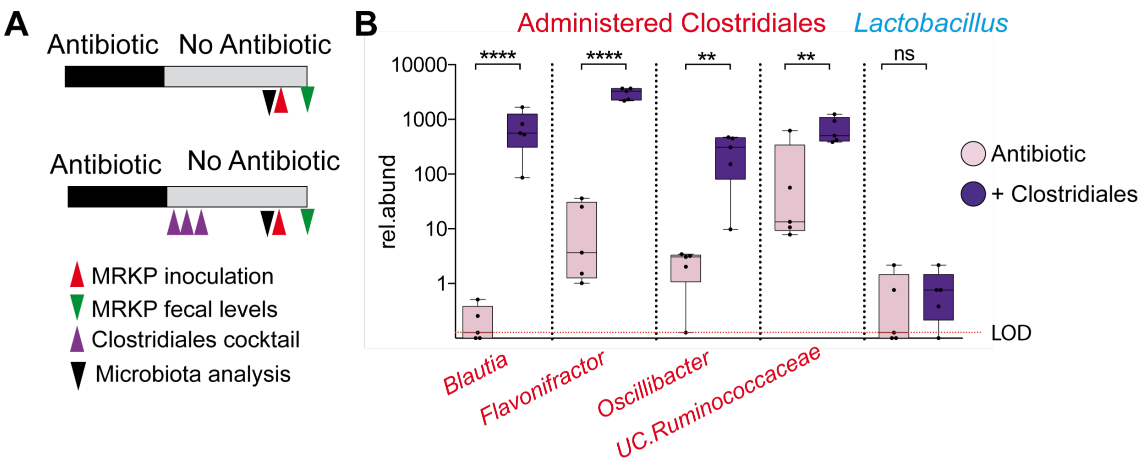

Supplementary Figure 11. Administration of a Clostridiales cocktail significantly increases the fecal levels of Clostridiales taxa included in the cocktail and does not change *Lactobacillus* levels. Schematic representation of the experiment in which we tested the effect of Clostridiales administration on multidrug-resistant *K. pneumoniae* (MRKP) gut colonization. Mice were treated with antibiotics (AVN) for a week in drinking water. After cessation of the treatment, one group was orally gavaged with a Clostridiales cocktail containing four Clostridiales isolates from the genera *Blautia*, *Flavonifractor*, *Oscillibacter* and Unclassified *Ruminococcaceae* for three consecutive days, while another group received the bacterial vehicle

(PBS-GC) instead. Two weeks after the end of the antibiotic treatment, and 11 days after the last oral gavage of the cocktail, a fecal sample was collected for microbiota analysis and mice were inoculated with MRKP. **(B)** Relative abundance (counts per 10000) of *Lactobacillus* or the Clostridiales taxa that were administered to mice. Two-sided Ancom2 test, ****p<0.0001, **p<0.01, ns- not significant: p=0.563, and Benjamini-Hockberg correction (q<0.05), N=5 mice per group. p and q values for all analyzed genera and other bacteria that could not be classified to the genus level (median >0.01%) are shown in Suppl. Data File 9. Rel.abund: relative abundance: counts per 10000 sequences. LOD: limit of detection. All Boxes extend from the 25th to 75th percentiles. The line within the boxes represents the median. Whiskers indicate the maximum and minimum values. Source data are provided as a Source Data file.

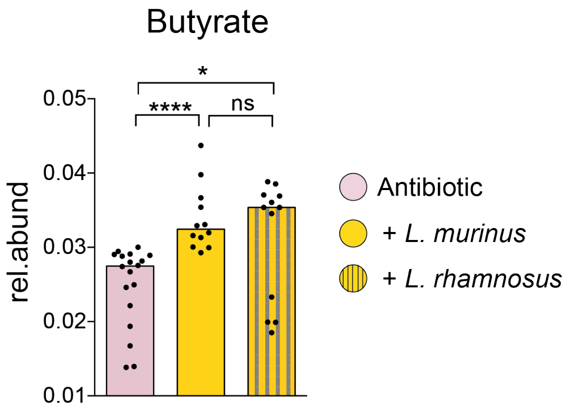

Supplementary Figure 12. Both *L. rhamnosus* and *L. murinus* increase the levels of butyrate in antibiotic-treated mice. Butyrate levels in the fecal samples collected from mice that received *L. murinus* or *L. rhamnosus* after antibiotic (AVN) cessation and those animals that received the bacterial vehicle (PBS-GC) instead. Two-sided Mann-Whitney test, ****p=1.6e-7, *p=0.027,ns- not significant: p=0.977, N=18, 12 and 12 mice per group. Bars represent the median. Rel.abund: relative abundance. Source data are provided as a Source Data file.

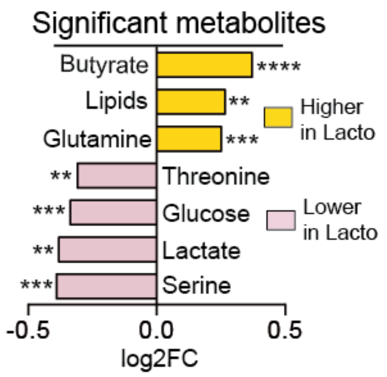

Supplementary Figure 13. Metabolites whose levels significantly differ in feces of the mice inoculated with *L. murinus* and *L. rhamnosus*, compared to un-inoculated animals. Metabolites were identified through NMR in murine fecal samples 2 weeks after stopping antibiotic (AVN) treatment and 11 days after bacterial gavage. Bars represent the log2 fold change (log2FC) between (i) the average levels detected in mice that received *Lactobacillus* as compare to (ii) the average levels detected in mice that did not received *Lactobacillus*. Only those metabolites that were found to be significantly different after both the administration of *L. murinus* and *L. rhamnosus* are shown. Two-sided Mann-Whitney test,****p=2.4e-5, ***p<0.001, **p<0.01, and Benjamini-Hockberg correction (q<0.05), N=18 and 24 mice per group. p and q values for all significant metabolites, including those only significant after the administration of either *L.murinus* or *L. rhamnosus* are shown in Suppl. Data File 10. Lacto: *Lactobacillus*. Source data are provided as a Source Data file.

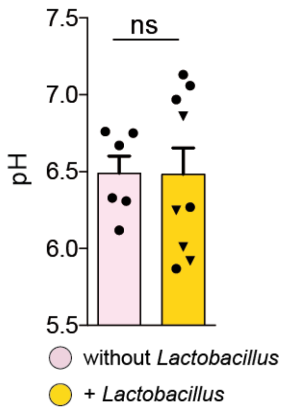

Supplementary Figure 14. pH in the murine caecum contents is unchanged after the administration of *Lactobacillus*. The pH found in the caecum of mice treated with antibiotics that received or not *Lactobacillus*. Triangles represent mice that received *L. rhamnosus*, while circles represent mice that received *L. murinus*. Two-sided T-test, ns- not significant: p=0.974, N=6 and 9 mice per group. Bars represent the mean. Whiskers represent the SEM. Source data are provided as a Source Data file.

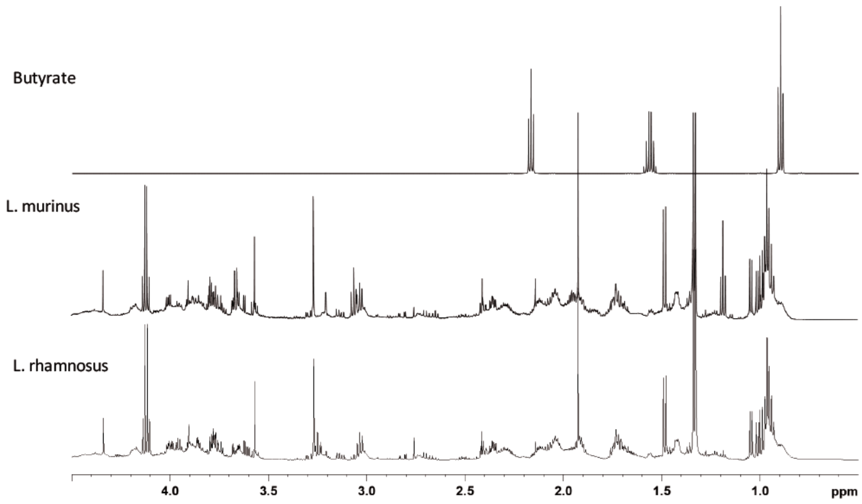

Supplementary Figure 15. *Lactobacillus* isolated strains do not produce butyrate *in vitro.* *Lactobacillus* culture supernatants were analyzed through NMR and their spectra was compared to that one of pure butyrate. We did not detect any clear signal with coupling pattern detected for pure butyrate (neither a triplet in 2.2 ppm, a sextuplet in 1.56 ppm nor a triplet at 0.89 ppm) in the *Lactobacillus* samples. These results suggest that these two *Lactobacillus* strains are not butyrate producers. (N= 1 sample per group)

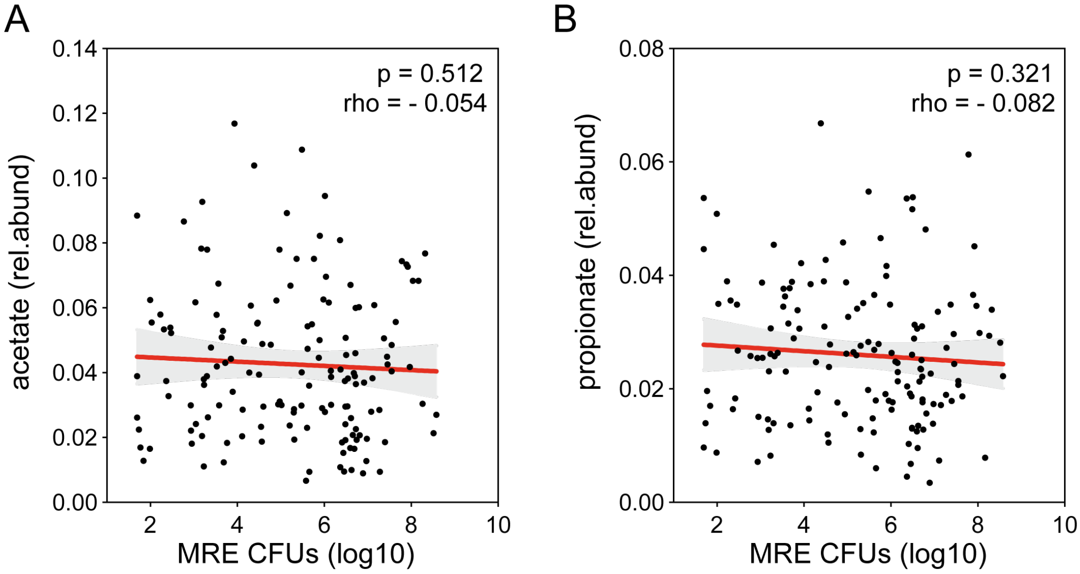

Supplementary Figure 16. Other major SCFAs are not significantly associated with multidrug-resistant *Enterobacteriaceae* (MRE) levels in acute leukemia patients. Correlation between acetate **(A)** or propionate **(B)** levels and MRE levels in MRE-colonized samples collected from hospitalized leukemia patients. Two-sided Spearman correlation test, p=0.512 for (A) and p=0.321 for (B), N=147 samples. The line represents the linear regression mean and the

grey shadow the 95% CI. Rel.abund: relative abundance. CFUs: colony forming units. Source data are provided as a Source Data file.

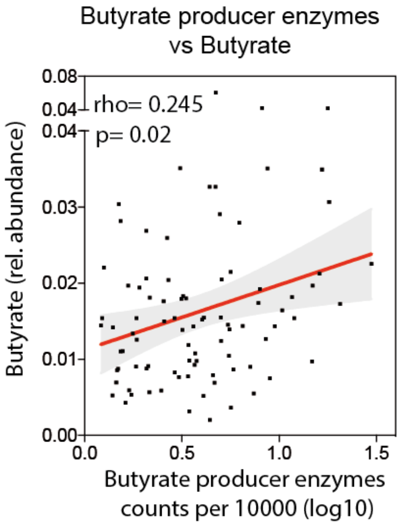

Supplementary Figure 17. Fecal levels of enzymes involved in production of butyrate are positively associated with the butyrate levels. Spearman correlation analysis of the fecal levels of enzymes involved in production of butyrate and the fecal levels of butyrate. Only samples containing detectable levels of enzymes involved in production of butyrate were included in the analysis (see methods). Two-sided Spearman correlation test, p=0.02, N=89 samples. The line represents the linear regression mean and the grey shadow is the 95% CI. Source data are provided as a Source Data file.
