# Supplementary material for: Lactobacillus supports Clostridiales to restrict gut colonization by multidrug-resistant Enterobacteriaceae
Source: Nat Commun. 2022 Sep 24;13:5617. doi: 10.1038/s41467-022-33313-w (PMC9509339; doi:10.1038/s41467-022-33313-w)
Supplement: Supplementary file 3 — Description of Additional Supplementary Files [file 41467_2022_33313_MOESM3_ESM.pdf]

Description of Additional Supplementary Files

Supplementary Data 1. Fecal samples and acute leukemia patients included in this study.

Supplementary Data 2. Prevalence and abundance of genera included in the analysis of human samples.

Supplementary Data 3. Correlation between the changes in the fecal levels of MRE in patients and the changes in the levels of genera or OTUs.

Supplementary Data 4. List of L. rhamnosus strains and ANI values.

Supplementary Data 5. Taxa that discriminate untreated mice from mice that were allowed to recover from antibiotic treatment.

Supplementary Data 6. Significantly different taxa between mice treated with antibiotics that received or not Lactobacillus.

Supplementary Data 7. Significantly different taxa between mice treated with antibiotics that received or not Lactobacillus identified by using sequencing data that has been normalized with 16S rRNA qPCR.

Supplementary Data 8. Correlation between the relative abundance of taxa and MRKP in mice.

Supplementary Data 9. Significantly different taxa between mice treated with antibiotics that received or not the Clostridiales cocktail.

Supplementary Data 10. Significantly different metabolites between mice treated with antibiotics that received or not Lactobacillus.

Supplementary Data 11. Correlation between the relative abundance of taxa and butyrate, serine, threonine or glucose in mice.

Supplementary Data 12. Correlations between the relative abundance of taxa, butyrate and Lactobacillus levels in patients.

Supplementary Data 13. Characteristics of the patients included in this study.

Supplementary Data 14. Tables containing the Omic processed data used in this study.
