# Supplementary material for: Lactobacillus supports Clostridiales to restrict gut colonization by multidrug-resistant Enterobacteriaceae
Source: Nat Commun. 2022 Sep 24;13:5617. doi: 10.1038/s41467-022-33313-w (PMC9509339; doi:10.1038/s41467-022-33313-w)
Supplement: Supplementary file 18 — Reporting Summary [file 41467_2022_33313_MOESM18_ESM.pdf]

nature portfolio

Corresponding author(s): Carles Ubeda

Last updated by author(s): Sep 7, 2022

Reporting Summary

Nature Portfolio wishes to improve the reproducibility of the work that we publish. This form provides structure for consistency and transparency in reporting. For further information on Nature Portfolio policies, see our Editorial Policies and the Editorial Policy Checklist.

Statistics

For all statistical analyses, confirm that the following items are present in the figure legend, table legend, main text, or Methods section.

n/a	Confirmed	
	☒	The exact sample size (*n*) for each experimental group/condition, given as a discrete number and unit of measurement
	☒	A statement on whether measurements were taken from distinct samples or whether the same sample was measured repeatedly
	☒	The statistical test(s) used AND whether they are one- or two-sided<br>*Only common tests should be described solely by name; describe more complex techniques in the Methods section.*
☒		A description of all covariates tested
	☒	A description of any assumptions or corrections, such as tests of normality and adjustment for multiple comparisons
	☒	A full description of the statistical parameters including central tendency (e.g. means) or other basic estimates (e.g. regression coefficient) AND variation (e.g. standard deviation) or associated estimates of uncertainty (e.g. confidence intervals)
	☒	For null hypothesis testing, the test statistic (e.g. *F*, *t*, *r*) with confidence intervals, effect sizes, degrees of freedom and *P* value noted<br>*Give P values as exact values whenever suitable.*
☒		For Bayesian analysis, information on the choice of priors and Markov chain Monte Carlo settings
☒		For hierarchical and complex designs, identification of the appropriate level for tests and full reporting of outcomes
	☒	Estimates of effect sizes (e.g. Cohen's *d*, Pearson's *r*), indicating how they were calculated

Our web collection on statistics for biologists contains articles on many of the points above.

Software and code

Policy information about availability of computer code

Data collection	H NMR spectra of fecal samples were obtained using a Bruker Avance III HD 600 MHz NMR spectrometer. 16S rRNA sequences were obtained using the MiSeq Illumina platform. Metabolic profile of Lactobacillus conditioned media was obtained using Agilent 8890 GC coupled to Agilent 5977B mass selective detector.
Data analysis	Quality of capillary electrophoresis obtained sequences was analyzed with Trev program from package Staden 2.0. Phylogenetic classification of the sequences was done using Mothur v.1.36.<br>High-throughput 16s rRNA sequencing analysis: quality assessment of the obtained MiSeq illumina sequences was performed using printseq-lite v.0.20.4. Pair-end sequences were assembled using fastq-join v.1.1.2. Assembled sequences were processed using Mothur v.1.36. Sequences were aligned against the 16S rRNA gene SILVA reference alignment with the Needleman-Wunsch algorithm implemented in Mothur v.1.36. Uchime algorithm implemented in Mothur v.1.36 was used to remove potentially chimeric sequences and vsearch algorithm implemented in Mothur v.1.36 was used to identify OTUs. Phylogenetic classification of sequences was performed with the Bayesian classifier algorithm implemented in Mothur v.1.36. Shannon index was obtained at the OTU level using the package vegan v2.5-3 and the R 3.4.0 software. PCoA analysis was performed with the R package labdsv 2.0.<br>Analysis of the prevalence of OTU20 (L. rhamnosus) in other cohorts worldwide: the representative sequence of the OTU20 (the most abundant sequence within this OTU) was aligned against each sequence downloaded from Flemish Gut Flora project (PMID: 30718848), MORINAGA and NIBIONH cohorts (PMID: 34016052), Milieu Intérieur project(PMID: 31519223) and the Spanish Gut Microbiome Project (PMID: 34759297) databases using blastN v.2.12.<br>Genome sequencing analysis: adaptor sequences were removed using Cutadapt v.1.10. Sequences were then filtered by quality using UrQt v.1.0.18. Cleaned genomic data was assembled using SPAdes v.3.7.1. Open reading frames (ORFs) were identified and annotated using PROKKA v.1.13. Genomes of interest were mapped against NCBI RefSeq database obtained core genomes using bowtie2 v.2.2.9. To reconstruct the Lactobacillus phylogeny a concatenate of the core-genes was built using the protein sequences, and aligned using mafft v.7407 with the –linsi algorithm, and the phylogenetic tree was reconstructed using iqtree2 v.2.1.0. Visualization of the tree was performed using FigTree v.1.4.3. ORFs annotation was performed using HMMer v.3.1.2

Metabolomic analysis of fecal samples: 1H NMR spectra of fecal samples was obtained using Topspin v.3.2 software. NMR spectra was manually corrected for phase and baseline distortions using Topspin v.3.5 software. NMR spectra was imported in the AMIX software v.3.9 for data processing.
Metabolomic analysis of Lactobacillus conditioned media: Peaks representing metabolites of interest were extracted using Matlab R2021b and an in-house script (available at https://github.com/anadj-micro/Analyzing-Lactobacillus-conditioned-media.git).
Statistical analysis were performed with Graphpad Prism 6.0, the R stats package v.3.6.0, LefSe, Ancom2, the adonis function of the R vegan package v2.5-3 and Fitlme function from Matlab R2021b. PCoA analysis that was performed with the R package labdsv 2.0.
All software used in this study is publicly available.

For manuscripts utilizing custom algorithms or software that are central to the research but not yet described in published literature, software must be made available to editors and reviewers. We strongly encourage code deposition in a community repository (e.g. GitHub). See the Nature Portfolio guidelines for submitting code & software for further information.

Data

Policy information about availability of data

All manuscripts must include a data availability statement. This statement should provide the following information, where applicable:
- Accession codes, unique identifiers, or web links for publicly available datasets
- A description of any restrictions on data availability
- For clinical datasets or third party data, please ensure that the statement adheres to our policy

16S rRNA sequencing data has been deposited in the Sequence Read Archive (NCBI) under accession code numbers: PRJNA870718 for murine fecal samples, PRJNA870756 for human fecal samples. Metabolomic data from fecal samples has been deposited in the EMBL-EBI MetaboLights database, with the identifier MTBLS5811. Metabolomics data from Lactobacillus secretome experiments have been deposited to the EMBL-EBI MetaboLights with the identifier MTBLS5733. Proteomic data was previously published (PMID: 30626002), and deposited in ProteomeXchange Consortium under accession number PXD011515, and the shotgun sequencing data for ORF identification has been deposited in the Sequence Read Archive (NCBI) under the accession number PRJNA877821. MRE levels and clinical patients data can be accessed in NCBI in the supplementary information of the previous published manuscript (PMID: 31767720) Tables containing the abundance of Genera, OTUs, KEGGs and metabolites from human samples are included as Supplementary material (Suppl. Data File 14). Tables containing the abundance of Genera and metabolites from murine fecal samples are also included as supplementary material in the Suppl. Data File 14. A table containing the abundances corresponding to metabolites detected in Lactobacillus secretome experiment is also included as Supplementary material in Suppl. Data file 14.
The following databases/datasets have been used in this manuscript: 16S rRNA SILVA reference alignment implemented in Mothur (https://mothur.org/wiki/silva_reference_files/), Mothur formatted RDP training set (https://mothur.org/wiki/rdp_reference_files/), NCBI RefSeq database (https://www.ncbi.nlm.nih.gov/refseq/), NIST library (https://www.nist.gov/programs-projects/tandem-mass-spectral-library), KEGG database (https://www.genome.jp/kegg/ko.html).
We also used the DNA sequences of the NIBIOHN cohort, which have been deposited in DDBJ under accession numbers DRA010837– DRA010841 (https://ddbj.nig.ac.jp/DRASearch/study?acc=DRP007218, DRP007219, DRP007220, DRP007221, DRP007222). The DNA sequences of the MORINAGA cohort, which have been deposited in DDBJ under accession numbers DRA009764 – DRA009767 (https://ddbj.nig.ac.jp/DRASearch/study?acc= DRP005906). The DNA sequences from the Flemish Gut Flora project sequence database (PMID: 30718848, available upon request), Milieu Intérieur project sequence database (PMID: 31519223, available upon request) and the Spanish Gut Microbiome Project sequence database (PMID: 34759297, available upon request).
Peaks representing metabolites of interest from the Lactobacillus conditioned media were extracted using Matlab R2021b and an in-house script available at https://github.com/anadj-micro/Analyzing-Lactobacillus-conditioned-media.git.
Source data are provided with this paper.

Field-specific reporting

Please select the one below that is the best fit for your research. If you are not sure, read the appropriate sections before making your selection.

☒ Life sciences ☐ Behavioural & social sciences ☐ Ecological, evolutionary & environmental sciences

For a reference copy of the document with all sections, see nature.com/documents/nr-reporting-summary-flat.pdf

Life sciences study design

All studies must disclose on these points even when the disclosure is negative.

Sample size	Sample-size calculation was not performed since it was not possible to predict the magnitude of the variation between animals for a particular parameter based on our current knowledge. However, considering previous studies on colonization resistance using SPF mice, at least 5 mice per group were included in all in vivo experiments with SPF mice. This allowed us to detect statistically significant differences among groups of mice regarding the major factors evaluated (pathogen gut levels, differences in microbial taxa and metabolites). For in vitro assays and the experiment in GFM, we detected a lower variability between samples from the same group (as compared to the variability detected in SPF mice). Since a lower number of mice is required to obtain statistically significant results when intragroup variability decreases, in these particular cases the number of samples that were included per group was at least 3. This number of samples allowed reaching statistically significant results when differences in the variable under study were detected among groups of samples.<br>For the patients' data, all available patients and samples that matched the inclusion criteria were included.
Data exclusions	In a previously published study we demonstrated that beta-lactam administration significantly reduced the fecal levels of multidrug-resistant Enterobacteriaceae (MRE) strains sensitive to the administered beta-lactam (PMID: 31767720). In order to avoid this confounding variable, for this work, we excluded those pairs of samples colonized with MRE isolates sensitive to the beta-lactams that were being administered to the patient during the collection of the samples.
Replication	Experiments were performed at least two times. When experiments were repeated, they yielded comparable results. The inhibitory effect of Lactobacillus against MRE colonization was validated in mice with two different Lactobacillus strains (from human and mouse origin). The

	negative impact of Lactobacillus, Clostridiales and butyrate on MRE murine intestinal colonization was validated through sequencing and metabolomic analysis in hospitalized patients
Randomization	All mice were randomized to avoid cage effects. For patients analysis, no randomization was required since patients were not divided into different groups.
Blinding	Blinding was applied to the quantification of the CFUs in order to determine the levels of MRE in the different groups of samples.<br>Also blinding was applied to the RNA and DNA extraction and sequencing and the metabolomic sample preparation. No blinding was used for the analysis of omic data since the previous knowledge of the groups of samples is required to perform the statistical analysis No blinding was applied in the in vitro assays since the results were obtained directly from the spectrophotometer.

Reporting for specific materials, systems and methods

We require information from authors about some types of materials, experimental systems and methods used in many studies. Here, indicate whether each material, system or method listed is relevant to your study. If you are not sure if a list item applies to your research, read the appropriate section before selecting a response.

Materials & experimental systems

n/a	Involved in the study
[x]	[ ] Antibodies
[x]	[ ] Eukaryotic cell lines
[x]	[ ] Palaeontology and archaeology
[ ]	[x] Animals and other organisms
[ ]	[x] Human research participants
[x]	[ ] Clinical data
[x]	[ ] Dual use research of concern

Methods

n/a	Involved in the study
[x]	[ ] ChIP-seq
[x]	[ ] Flow cytometry
[x]	[ ] MRI-based neuroimaging

Animals and other organisms

Policy information about studies involving animals; ARRIVE guidelines recommended for reporting animal research

Laboratory animals	All experiments with SPF mice were done with 7-week-old C57BL/6J female mice purchased from Charles River laboratories and housed with autoclave-sterilized food (a 1:1 mixture of 2014S Teklad Global diet and 2019S Teklad Global Extruded 19% Protein Rodent Diet from Envigo) and autoclave-sterilized water. Temperature is kept at 21ºC +/- 2ºC and humidity is maintained at 60-70%, in 12h light/dark cycles.<br>All gnotobiotic experiments were done with 7-week-old C57BL/6J female mice and were performed under aseptic and sterile conditions. Animals are maintained singly housed with food and water ad libitum inside airtight HEPA-filtered isocages placed in an isocage rack. Temperature is kept at 22ºC +-2ºC and humidity is maintained at 45-50% +-10%, in 12h light/dark cycles. Animal manipulation is performed in an ISOcage Biosafety Cabinet, under sterile conditions. Mice were fed with a rat and mouse number 3 breeding autoclave diet from Special Diet Services.
Wild animals	The study did not involve wild animals.
Field-collected samples	The study did not involve samples collected from the field
Ethics oversight	All mouse procedures including SPF mice were performed in accordance with institutional protocol guidelines at the "Servei Central de Suport a la Investigació Experimental" at the University of Valencia. Mice were maintained accordingly to the National guidelines (RD 53/2013), under protocols approved by University of Valencia Animal Care Committee describing experiments specific for this study.<br>All mice procedures with GFM were performed at Instituto Gulbenkian de Ciência (IGC) and were approved by the Institutional Ethics Committee and the Portuguese National Entity (Direção Geral de Alimentação e Veterinária; Ref. number 015190), which complies with European Directive 86/609/EEC of the European Council.

Note that full information on the approval of the study protocol must also be provided in the manuscript.

Human research participants

Policy information about studies involving human research participants

Population characteristics	Hospitalized patients diagnosed with acute leukemia. Age and sex of the patients included in the analysis are indicated in the Supplementary Data file 13. Other covariate population characteristics are published as a part of the study by Djukovic et al., AAC, 2020 (PMID31767720).
Recruitment	All the acute leukemia patients to be admitted to Hospital La Fe (Valencia, Spain) from December 2013 until May 2015, and who agreed to participate in the study, were enrolled. Because the colonization with MRE during the hospital stay cannot be predicted ahead of the hospital admission, patients didn't have strong personal reasons to participate or not in the study at the time of admission, thus there was no obvious self-selection bias.
Ethics oversight	The study involving patients was conducted in accordance with the Declaration of Helsinki, and the protocol was approved by

the Ethics Committee of CEIC Dirección General de Salud Pública y Centro Superior de Investigación en Salud Pública (20130515/08). All included patients gave their consent to participate in the study. Participants did not receive any compensation for participating in the study.

Note that full information on the approval of the study protocol must also be provided in the manuscript.
